# Recent advances in GTR scaffolds

**DOI:** 10.6026/973206300181181

**Published:** 2022-12-31

**Authors:** Devika Bajpai, Arvina Rajasekar

**Affiliations:** 1 Department of Periodontology, Saveetha Dental College and Hospitals, Saveetha Institute of Medical and Technical Sciences, Saveetha University, Chennai - 600077

**Keywords:** Periodontitis, GTR membrane, scaffold, electro spinning, 3D printing

## Abstract

Periodontitis is a serious chronic inflammatory condition that can cause periodontal tissue deterioration and, eventually, tooth loss. Periodontal regenerative therapy using membranes and bone grafting materials, as well as flap debridement and/or flap curettage, have all been used with varying degrees of clinical effectiveness. Current resorbable and non-resorbable membranes serve as a physical barrier, preventing connective and epithelial tissue down growth into the defect and promoting periodontal tissue regeneration. The "perfect" membrane for use in periodontal regenerative therapy has yet to be created, as these conventional membranes have several structural, mechanical, and bio-functional constraints. We hypothesised in this narrative review that the next-generation of guided tissue and guided bone regeneration (GTR/GBR) membranes for periodontal tissue engineering will be a graded-biomaterials that closely mimics the extracellular matrix.

## Background:

Periodontitis is one of the most devastating disorders, affecting the periodontal system's integrity and leading to periodontal tissue degradation and, eventually, tooth loss [1]. Clinical and/or surgical interventions are widely established to be necessary for the restoration of periodontal tissue health. Various regenerative surgical techniques for the regeneration of periodontal specific tissues, including as alveolar bone, cementum, periodontal ligament, and gingiva, have been suggested and investigated over the last decade [2 -3,4]. Periodontal tissue regeneration, for example, has had a lot of success when GTR/GBR techniques have been used in particular, well-selected clinical patients. Nonetheless, outcomes vary according on the patient's age, defect size, generics, and other demographic and lifestyle factors. Innovations in science and technology of nano material have increased popularity for techniques such as electrospinning of biomimetic multifunctional growth enhancing regenerative membranes. In this narrative review recent advances in GTR membrane and it's future perspective has been discussed.

## Barrier membrane for GTR and GBR applications:

GTR/GBR membranes were developed as a result of a strategy to isolate the periodontal defect with a mat-like material (resorbable or non-resorbable) that would act as a physical barrier to prevent gingival cell invasion. These GTR/GBR membranes must have the following properties: (1) biocompatibility to allow integration with host tissues without inducing inflammatory responses, (2) proper degradation profile to match that of new tissue formation, (3) adequate mechanical and physical properties to allow placement in vivo, and (4) sufficient sustained strength to avoid membrane collapse and perform their barrier function [5, 6,^7^, ^8^, ^9^, ^10^, ^11^]. According to their degrading characteristics, GTR/GBR membranes are categorised into two groups: non-resorbable and resorbable.

## Stability and degradation characteristics of GTR/GBR membranes:

The so-called "gold standard" non-resorbable membranes for GTR/GBR procedures currently on the market are high-density poly tetra fluoro ethylene, PTFE. PTFE membranes are inert and biocompatible, act as a cellular barrier, provide space for tissue regeneration, and allow tissue integration. It has been suggested that there is a favourable correlation between the level of bone regeneration and space protection [12, 13]. Studies have revealed that titanium reinforcement of high-density PTFE membranes lead to superior regenerative capacity when compared to traditional expanded PTFE membranes mainly due to the additional mechanical support provided by the titanium frame against the compressive forces exerted by the overlying soft tissue. The majority of synthetic polymer resorbable membranes for periodontal regeneration on the market are either based on polyesters (e.g., poly(glycolic acid) (PGA), poly(lactic acid) (PLA), poly(e-caprolactone) (PCL), and their copolymers) [14 16] or tissue-derived collagens. The polyester-based membranes are biocompatible, biodegradable, and easier to handle clinically when compared to PTFE membranes as well as allowing tis- sue integration. Their resorption rate is important since these membranes must function for at least 4-6 weeks to allow successful regeneration of the periodontal system. Collagen is a prominent component of the extracellular matrix of the body (ECM). However, because of the high cost and low definition of commercial sources, type-I collagen may have restrictions in its application, making it difficult to control degradation and mechanical qualities. AlloDerm® is a type-I collagen-based acellular freeze-dried dermal matrix graft generated mostly from human cadaveric skin. In vivo, collagen-based membranes have exhibited poor performance as the membrane degrades.[17, 19, 20]

## Designed membranes for zone-dependent bioactivity:

Many research groups have attempted to build and produce GTR/GBR periodontal membranes with the required features and properties using a combination of natural and synthetic polymers in recent years. Film casting, dynamic filtration, and e-spinning [21, 22,23, 24,25] of synthetic (e.g., PCL) and/or natural (e.g., collagen, chitosan) polymers were used to create GTR/GBR membranes in this research.

## Membrane electro spinning (e-spinning):

Electrospinning, often known as e-spinning, was first introduced by Formhals in 1938. Several research groups have recently investigated its usage in the creation of fibrous scaffolds for tissue regeneration [26 27 28 29 30]. A polymer solution/melt in a syringe is charged by a high voltage source, and a grounded plate is positioned at a predetermined distance from the needle tip in standard electro-spinning equipment.

## Nano-composite electrospun fibers in periodontal regeneration:

Various types of nano-components are:

## Nano-composite electrospun fibers blended with polymer matrixes:

Synthetic polymers have strong mechanical and electrospinnability, but they have low biological properties. It's a promising technique to combine natural polymers with built-in bioactivity with synthetic polymers to enhance cellular reactions in periodontal regeneration. Polysaccharides, such as chitosan, cellulose, and alginate, are popular in tissue engineering because of their excellent biological characteristics and ease of use.

[1] Nano-Composite Electrospun Fibers Blended With Inorganic Components

[2] Nano-Composite Electrospun Fibers Blended With Ceramic Components

1) Blended with Ca-P based components

2) Blended with Ca-Si based components

3) Blended with oxide components

[3] Nano-Composite Electrospun Fibers Blended With Metal Components

1) Various metal nanoparticles with different characteristics can be incorporated into nanofibers to improve membrane properties like antibacterial activity and bone regeneration activity.

2) Like silver nanoparticles and gold nanoparticles.

## Nano-composite electrospun fibers blended with drugs, growth factors and proteins:

Drugs like metronidazole, ampicillin, amoxicillin, tetracycline hydrochloride, doxycycline hydrochloride and tinidazole and NSAIDs like ibuprofen and piroxicam. Growth factors like bone morphogenetic protein (BMPs) and platelet-derived growth factors (PDGF).

Antimicrobial peptides (AMPs) have a broad spectrum of antibacterial activity, distinguished from conventional antibiotics, which may result in bacterial resistance.

## 3D printed scaffold:

These innovative techniques use CAD and CAM technologies to 3D-print a desired structure based on a CAD file that already defines the exact dimensions of a scaffold. In a typical clinical case scenario, CAD models are created based on pictures from computed tomography (CT) scans of a patient-specific bone defect to design a "custom-made" bone graft substitute that could be beneficial in repairing lesions with complex geometry. With the growing demand for "optimal" tissue regeneration, "3D-printed" scaffolds have been tested in a variety of periodontal applications, including guided bone regeneration (GBR), guided tissue regeneration (GTR), vertical bone augmentation, sinus augmentation, and socket preservation, with mixed results.

## Scaffold seeded with multiple types of cells:

A feasible method for increasing the efficacy of integrated periodontal regen- eration is to seed single or multiple cells directly into the multi-phasic scaffold. Periodontal ligament stem cells (PDLSC) and bone marrow mesenchymal stem cells (BMMSC) are the most commonly used cells in integrated periodontal tissue regeneration because they are directly involved with target tissues.

## Smart scaffold constructs with stem cells for bone tissue engineering:

The three essential parts of bone tissue engineering are scaffolds, cells, and growth factors [31]. Scaffolds can be used as a delivery vehicle for cells and a carrier for growth factors in addition to replacing the extracellular matrix (ECM) [32]. Scaffolds have an effect on seeded cells, altering cell adhesion, migration, and proliferation, and hence regenerative medicine efficacy [33]. Bioactive compounds and nanoparticles have been incorporated into smart scaffolds, as well as specific alterations to the physical and chemical properties of the scaffolds [34]. They can increase cell connections by promoting osteogenic differentiation for bone healing and responding more effectively to the host environment.

## Biomimetic and bionic smart scaffolds:

Biomimetic smart materials are an important category of smart materials. Their design is based on the structure, function, and production of biological materials, which are biologically inspired [35]. In order to generate native tissue-like biomaterials, multifunctional and adaptive cellular microenvironments must be engineered. In tissue regeneration, the cell-biomaterial interface is a complex and dynamic milieu [36]. Smart scaffolds can generate the required cell responses by allowing stem cells in contact with the scaffold to perceive diverse qualities such as stiffness and nanostructure and respond appropriately. Using biomimetics, nano-assembly technology, and additive manufacturing techniques, smart artificial bone scaffolds were recently created to match the composition and structural features of genuine bone.

## Immune-sensitive smart scaffolds:

In vivo, however, scaffolds with low biocompatibility can cause severe foreign-body reactions. It is critical to develop smart immune modulatory biomaterials capable of directing the host response toward tolerance of foreign scaffolds or regulating immunological microenvironments to promote cell survival in order to avoid or reduce the potential immunological response between the host immune system and foreign scaffolds. The immune system is the host's first response, and it is crucial in responding to tissue trauma and biomaterial implantation.

## Shape-memory smart scaffolds:

Another type of smart scaffold is one that can remember its shape. An external stimulation, such as a temperature change [37], an electric or magnetic field [38- 39] or light, can cause shape-memory polymers (SMPs) to return to their original shape [40]. Because of their uses in tissue engineering, they've gotten a lot of attention [41]. The shape-memory behaviour of the scaffolds allows them to be predesigned, distorted for easy transplant into bone defects via minimally invasive surgery, and then extended to conform to an uneven bone defect [42, 43, 44, 45,46, 47]. The initial implant is tiny in size and can be implanted in the body utilising minimally invasive techniques that cause the least amount of damage to the host tissues. After being implanted, the implant expands to fill the bone deficiency.

## Electromechanical-stimulus smart scaffolds:

In addition, electromechanical-stimulus scaffolds are an important type of smart materials. The discovery of electric fields in biological tissues has resulted in the creation of therapies that use electrical stimulation [48]. The capacity of certain materials to create an electric charge in response to mechanical stress is known as the piezoelectric effect [49]. Certain crystals and ceramics, as well as some living tissues (such as natural bone, tendon, ligaments, cartilage, skin, dentin, and collagen) and biological macromolecules, are examples of piezoelectric materials (such as proteins, nucleic acids and muco poly saccharides). Neuromuscular activity, glandular secretion, cell membrane function, and tissue growth and repair are all influenced by biological electric fields in host tissues. [50] Efforts to produce smart electrically active biomaterials and scaffolds were made, with promising results.

## Summary and future perspective:

Periodontitis is a chronic inflammatory disease that can compromise the integrity of tooth support and, in the worst-case scenario, result in tooth loss. [51] Flap debridement and/or flap curettage, as well as periodontal regenerative therapy using membranes and bone grafting materials, have all been used with varying degrees of clinical effectiveness to date. Current resorbable and non-resorbable membranes serve as a physical barrier, preventing connective and epithelial tissue down growth into the defect and promoting periodontal tissue regeneration. The structural, mechanical, and bio functional constraints of traditional membranes are numerous. The "perfect" membrane for periodontal regeneration therapy has yet to be discovered [52- 54]. We hypothesised that a physiologically active, spatially planned, and functionally graded nano fibrous material that closely replicates human skin could be developed using a graded-biomaterials approach. Both physiologically active, functionally graded e-spun nano matrix and hydrogel combinations show substantial potential for use in periodontal tissue engineering, according to current technology and results from the literature. The rational design of hydrogels, which requires not only control of degradation and mechanical properties, but also consideration of biological aspects, is undoubtedly one of the future areas. Although the in vitro results with e-spun scaffolds are promising in terms of developing a synthetic biologically active membrane, these scaffolds have a severe drawback. Because of the dense packing of e-spun nano fibers during the spinning process, the resulting matrix has pore diameters that are typically small enough to allow cell infiltration into the bulk [55] which restricts tissue in-growth and proliferation. Although approaches like the salt leaching method [56] and selective removal of soluble sacrificial fibres can promote cell infiltration in fibrous mats, they can also compromise structural and mechanical integrity or cause macroscopic layer delamination. The implant must support the infiltration of bone cells from the bone defect side in order to enhance periodontal healing. Additionally, vascularization of the biomaterial/cell construct is an important phase in tissue healing because it delivers the nutrition and oxygen that bone cells require while also allowing cell waste products to be removed. As a result, in order to speed up the transition from bench to chairside for newly developed periodontal regeneration therapies, in vivo pre-clinical implantation testing to analyse the behaviour of barrier membranes in animal models should be the next step. [57] To figure out their genuine biomechanical integrity, biodegradation, healing, and vascularization properties, as well as regeneration and remodelling Material scientists, stem cell biologists, and fundamental and clinical dental researchers will need to work together closely to achieve this.
